# Pathological characterization of keel bone fractures in laying hens does not support external trauma as the underlying cause

**DOI:** 10.1371/journal.pone.0229735

**Published:** 2020-03-09

**Authors:** Ida Thøfner, Hans Petter Hougen, Chiara Villa, Niels Lynnerup, Jens Peter Christensen

**Affiliations:** 1 Department of Veterinary & Animal Sciences, University of Copenhagen, Copenhagen, Denmark; 2 Department of Forensic Medicine, University of Copenhagen, Copenhagen, Denmark; Tokat Gaziosmanpasa University, TURKEY

## Abstract

Keel bone fractures in laying hens have been described with increasing prevalence from several countries over the last twenty years and are considered one of the greatest welfare problems to the layer industry. In Denmark we have observed fracture prevalence in the range of 53% to 100% in flocks from cage-free systems whereas flock prevalences in birds from enriched cages ranged between 50–98%.

Previous research have speculated that the underlying reason for the development of keel bone fractures is trauma in relation to impact of the bird with furniture, other equipment etc. However, little evidence of this theory has been provided. Predisposing factors have also been suggested including genetics of the bird, lack of specific feedstuff components, high egg production, management factors and layer fatigue.

This study has addressed the possible pathogenesis of these fractures by pathological characterization of fractures in birds from different production systems. More than 60 keel bones with fractures have been characterized histo-pathologically and by CT scan. This included an assessment of damage to muscles and soft tissues, the bone and the healing process including callus formation. This investigation has shown that high energy collisions cannot be responsible for the majority of fractures, located at the caudal tip of the keel bone, observed in laying birds as markers associated trauma were not observed in the majority of the cases just as few recognized healing processes were observed. These results suggest an alternative pathogenesis to trauma.

## Introduction

Keel bone fractures in laying hens have been described with increasing prevalence from several countries over the last twenty years and are considered one of the greatest welfare problems to the layer industry [1,2]. All types of production system experience the problem although cage-free systems have been reported to have the highest occurrence of the problem. Prevalences in non-cage systems have been reported to range from 70 and up to 97% [3–8]. However, high prevalences, up to 62%, also has been reported from enriched cages [7,9]

Early reports have not distinguished between keel bone damage/deformities and fractures, but recent investigations have been more precise in separating these pathological conditions. The current opinion appears to be that deviations/deformities to a large extent is due to perching behavior in combination with hard, thin perches whereas fractures are the result of impact collisions with housing structures [10,11,5,12–14].

The possible impact of keel bone fractures, in particular, on production and welfare parameters have received quite a lot of attention by different research groups. In general, keel bone fractures are considered to be painful and reduce mobility [15–19]. The impact on production parameters appear to be less documented, however, increased feed conversion and reduced laying and egg quality have been reported [15–18,20]. In addition, several studies have focused on diagnosing the condition, mainly, by palpation techniques and radiographs [4,21–24].

Previous research have speculated that the underlying reason for the development of keel bone fractures is trauma in relation to impact of the bird with furniture, other equipment etc. [25,26]. However, little evidence of this theory has been provided. Predisposing factors have also been suggested including genetics of the bird, lack of specific feedstuff components, high egg production. Layer fatigue or poor bone health have also been suggested to be contributing factors [25,27,28].

Gross pathology of keel bone fractures often do not indicate trauma and the condition appears with high prevalence in enriched cages where trauma resulting in up to eight to ten fractures in one bird (own observation) is hard to understand. Hence, it seems obvious to assess the fractures pathologically and histopathologically to try to understand the development of these. Thus, the aim of the study was to observe if any “state of the art” used histological methods could reveal aspects of the unknown pathogenesis of the fractures and to see whether the characterization of the fractures would support the theory of trauma as the underlying cause. By combined CT scanning and different histopathological staining techniques the nature of the fractures is described.

## Materials & methods

The collected keel bones from end of lay (>75 weeks old) birds was selected from keel bones originating from a large study on keel bone fractures (not yet published). In brief, in this study we received 120 dead birds per flock culled at end of lay on different farms farm, representing both caged and cage-free birds.

**Fig 1:**
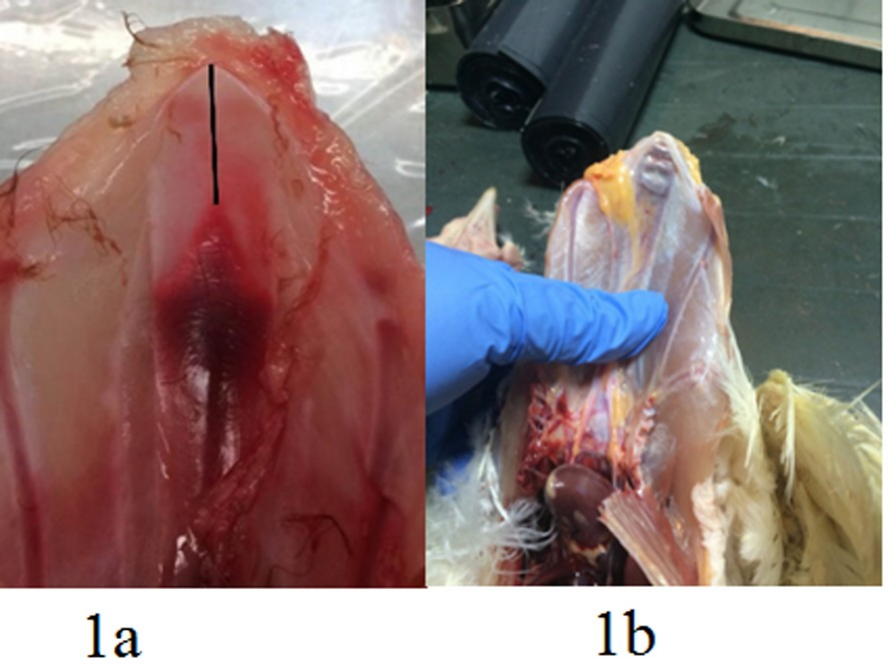
a: Illustration of measurement of non-ossified region at tip of the keel (cartilage) (black bar). Visceral (dorsal) aspect of the keel bone plate and b: typical location of fractures and with substantial callus formation.

Additionally, 74 keel bones from 32 weeks old layers at peak of production were collected and examined for presence of fractures and ossification status of the keel. Keel length, length of not yet ossified cartilage (Fig 1A) and fracture localization were measured with a digital caliper. Thirteen of these keel bones (3 with no fracture and 10 with fractures, Table 1) were further selected for detailed description of the fracture morphology by CT scan and histology.

**Table 1:** Overview of macroscopic fracture characteristics in birds selected for CT-scan and histomorphological fracture characterization.

Age	No. of birds	No fracture	Fresh fracture	Non-productive fracture	Productive fracture
>75 weeks	49 (14^a^/35^b^)	2^c^ (0/2)	6 (3/3)	23 (10/13)	18 (1/17)
32 weeks	13 ^b^	3	5	5	-

^a^ Caged birds.

^b^ Cage-free birds.

^c^ male birds.

Regardless of age the macroscopic appearance of the fractures formed the base for selection of keel bones for the present study in order to represent the most often observed fracture types in Denmark and representing different types of production systems; cages and cage-free. Macroscopic selection criteria was: pronounced callus formation (very common in cage-free systems); no or minimal callus formation (primarily seen in caged birds); and no fractures. The keels from birds selected for this study were dissected from the rest of the hens body with all the pectoral muscles and their fasciae left intact. The keel bones selected for histomorphological characterization and CT scan in the present work are thus partly a subpopulation of the large study on end of lay (>75 weeks old) and (49 keel bones) and selected keel bones from hens at peak production (32 weeks old) (13 keel bones). In total, 62 keel bones (49 from end-of-lay hens and 13 from 32 week old birds) were CT scanned and investigated histopathologically after they had been characterized macroscopically.

### The groups of birds

The old birds were end-of-lay hens which had been culled on-farm and subsequently collected by us. The 32 week old birds had been culled on-farm for various reasons and stored in a freezer and subsequently shipped to us. Consequently, there has been no need for approval by the Danish Animal Experiments Inspectorate to perform these post mortems. Participating farmers all approved the investigation. All birds were fed commercial layer feed.

### CT scanning

CT scanning of 62 keel bones with remaining soft tissues (intact muscles and fasciae) was performed using a Siemens Somatom Definition CT scanner with the following settings: 120 kV, 300 mAs, slice thickness 0.6cm, pitch 0.35 cm, slice increment 0.2 cm and a sharp reconstruction algorithm (H70h). A constant field of view of 17cm was used, resulting in a pixel size of 0.34 cm.

Mimics software, version 18 (Materialise Inc., Leuven, Belgium), was used to visualize the CT images and to generate 3D models of the keen bones. 3D models of the bone were generated using automatic segmentation applying a Hounsfield unit (HU) range from 226 to 3071.

**Fig 2:**
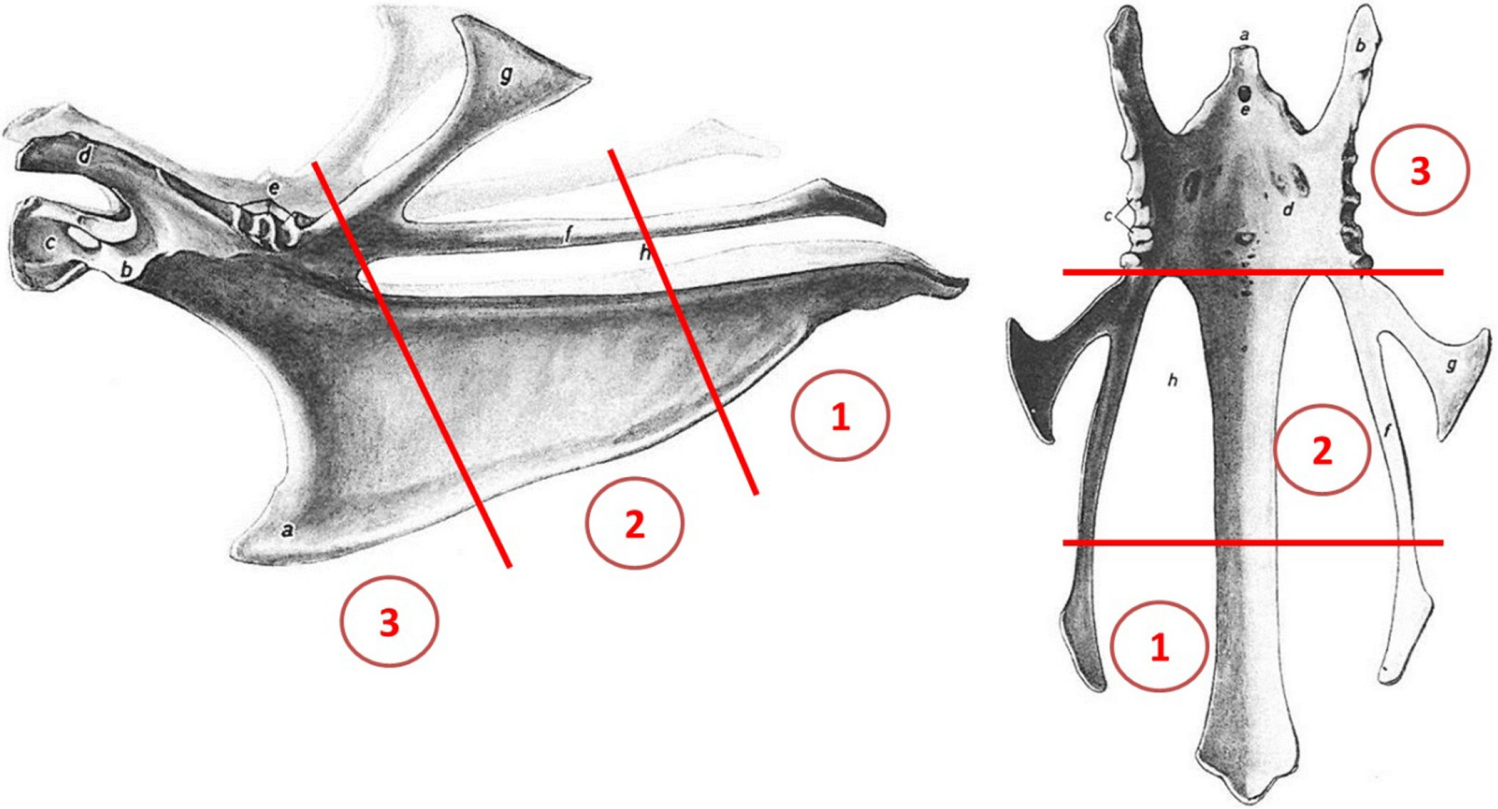
Lateral and dorsal (visceral) aspect of the keel bone of the adult chicken. Sections of the keel bone 1) the caudal part (including the tip, processus xiphoideus); 2) the middle part; and 3) the cranial part (including carina sterni). Modified after [29].

The assessor carrying out both the CT scans, 3D modelling and evaluation of the observations obtained was blinded to any information related to both origin, age, and macroscopic findings. The fractures were assessed both from the CT images and the 3D models. For localization of the fractures the keel was divided into three sections (Fig 2) [29]

### Histopathology

Immediately after CT scanning, all 62 keel bones including their intact pectoral muscles were fixed *in toto* in 4% neutral buffered formaldehyde. After demineralization with formic acid the caudal half of the keel bone with adjacent pectoral muscle were split in the sagittal plane (abaxial to the keel), both sections were mounted in large tissue cassettes and then dehydrated, decalcified in formic acid and embedded in paraffin wax and cut into 4 *μ*m thick sections. Hematoxylin and eosin (HE) staining was performed according to a standard protocol [30]. To evaluate presence of blood residues Perls’ Prussian blue stain for ferric iron was performed [31]. Collagen and callus formation were evaluated using Masson trichrome stain [31], whereas Safranin O staining was applied to distinguish between mature and immature osteoid in the fracture lines and the callus [32]. All slides were systematically semi-quantitatively evaluated for presence of fractures and soft tissue damage. The assessor was blinded to any information related to both origin, age, macroscopic findings as well as CT scanning observations. Presence and amount (absent (-), minimal (+), moderate (++), abundant (+++)) of hemorrhage, inflammation, fibrosis, ruptures in the soft tissue (i.e. muscles and fascia) were recorded. The fractures were evaluated for localization of fracture line (i.e. where it breached/severed the periost (ventral, visceral (dorsal) aspect or both aspects of the keel bone plate), degree of branching of fracture lines (i.e. simple or complex), width of fracture line at the periost (i.e. wide or narrow). Furthermore, the presence of hemorrhage, inflammatory cells, collagen, callus and maturity of the callus (i.e. immature or mature/calcified) were recorded similar to the soft tissue observations.

### Statistical analysis

For statistical analysis of the data, Fisher’s exact test, Chi square and z-test for multiple comparison between factors (e.g. age, housing system etc.) within a variable (e.g. fracture localization, remodeling state etc.) was performed in SPSS Statistics 25® (IBM Corporation). Descriptive statistics and t-test for comparing means was performed using Prism 8 for Windows (GraphPad Software, Inc.). Significance levels were set to *P*<0.05.

## Results

In total 62 keel bones, of which 57 keel bones presented visible and palpable fractures, were selected for further assessment based on the macroscopic appearance. They included 23 productive fractures (pronounced callus formation) (Fig 1B) and 17 non-productive fractures (no or minimal callus formation (Table 1.). The vast majority of these were located at the caudal tip of the sternum (last 5 cm of the keel) (Table 1.). In the group of 32 week old birds 27/74 had visible fractures, 15 had one fracture, five birds had two fractures, two birds had three fractures and five birds had 4 visible fractures. In eight of the birds one fracture was regarded as fresh due to visible, however, limited, hemorrhage in the fracture line. Measurements of keel length, length of ossified keel and cartilage length at caudal tip the keel did not reveal any statistical difference between birds without fracture and birds with fractures (t-test, *P*>0.05) (Table 2). Fractures (n = 51) were localized 26.3±7.3 mm (range of 12.1–51.3) mm from the caudal tip of the keel.

**Table 2:** Overview of keel length, ossification status and localization of fracture in 32 weeks old birds^a^.

	No. of birds	Total keel length (mm)	Length of ossified keel (mm)	Length of cartilage (mm)	Distance bone-cartilage rim to nearest fracture (mm)
No fractures identified	47	117.7±4.1	111.5±4.3	6.2±1.6	N/A
≥1 fracture identified	27	116.6±3.9	110.1±4.4	6.5±1.6	16.4±5.8

^a^ Cage-free birds.

### CT scans

**Fig 3:**
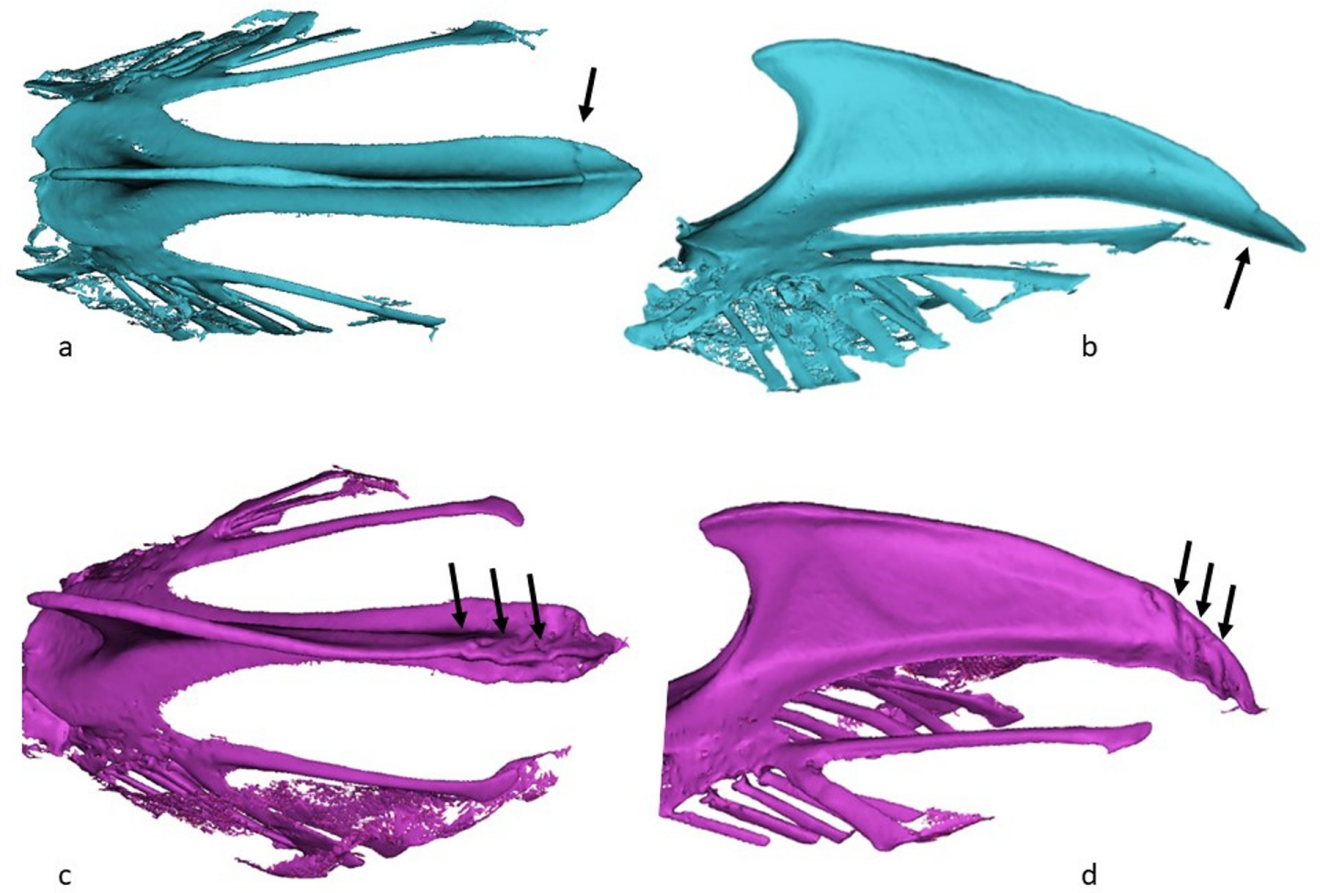
3D model from CT scanning of keel bones: a,b, frontal and lateral view of the keel bone of a 32-week hen (barn floor) showing one fracture (arrow); c, d, frontal and lateral view the keel bone of a 85-week hen (cages) showing minimum three fracture (arrows).

**Fig 4:**
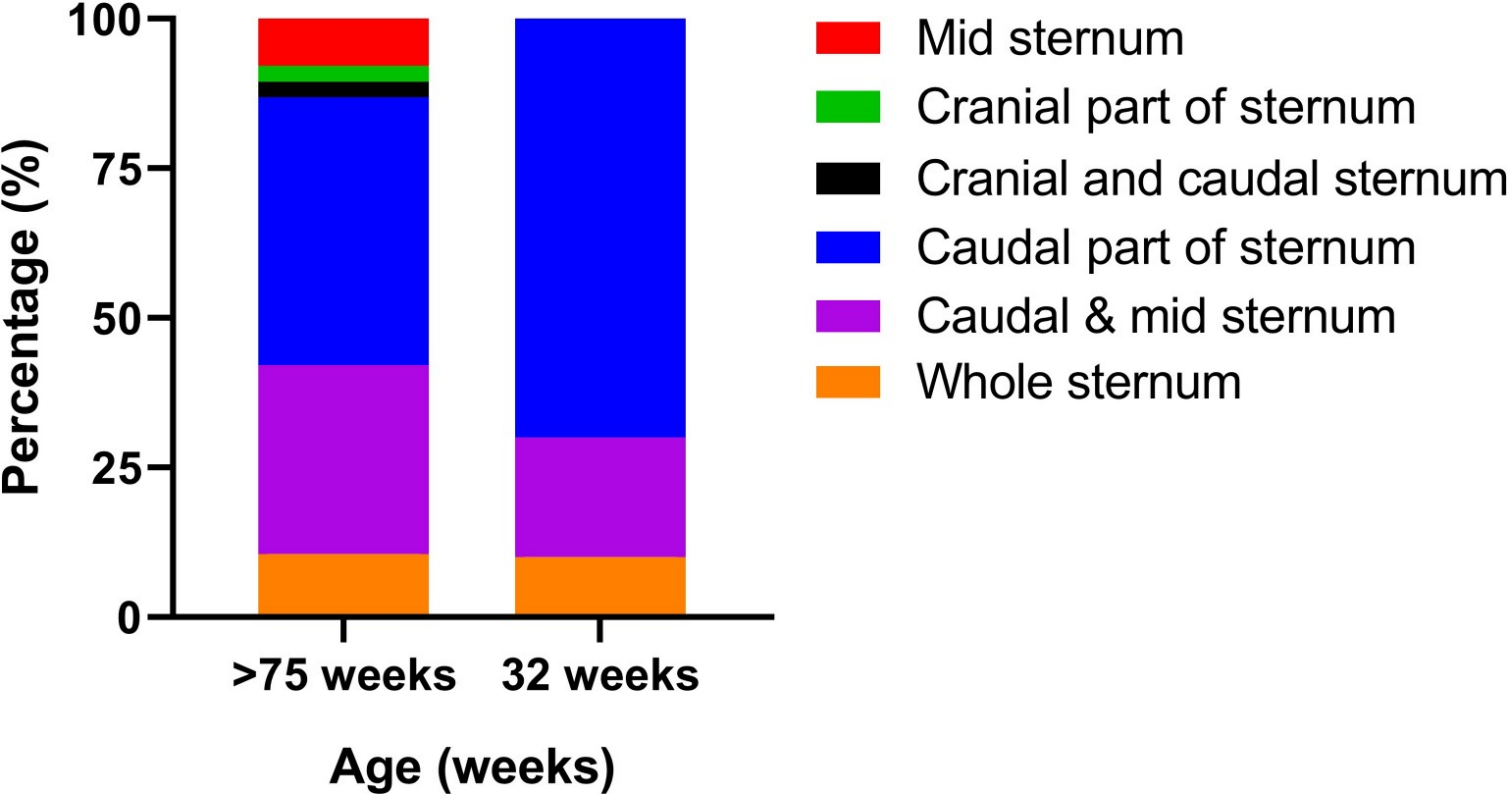
Distribution of the localization of fractures identified by CT scan in old (n = 38) and young birds (n = 10). Data is presented as percentage within age group.

**Fig 5:**
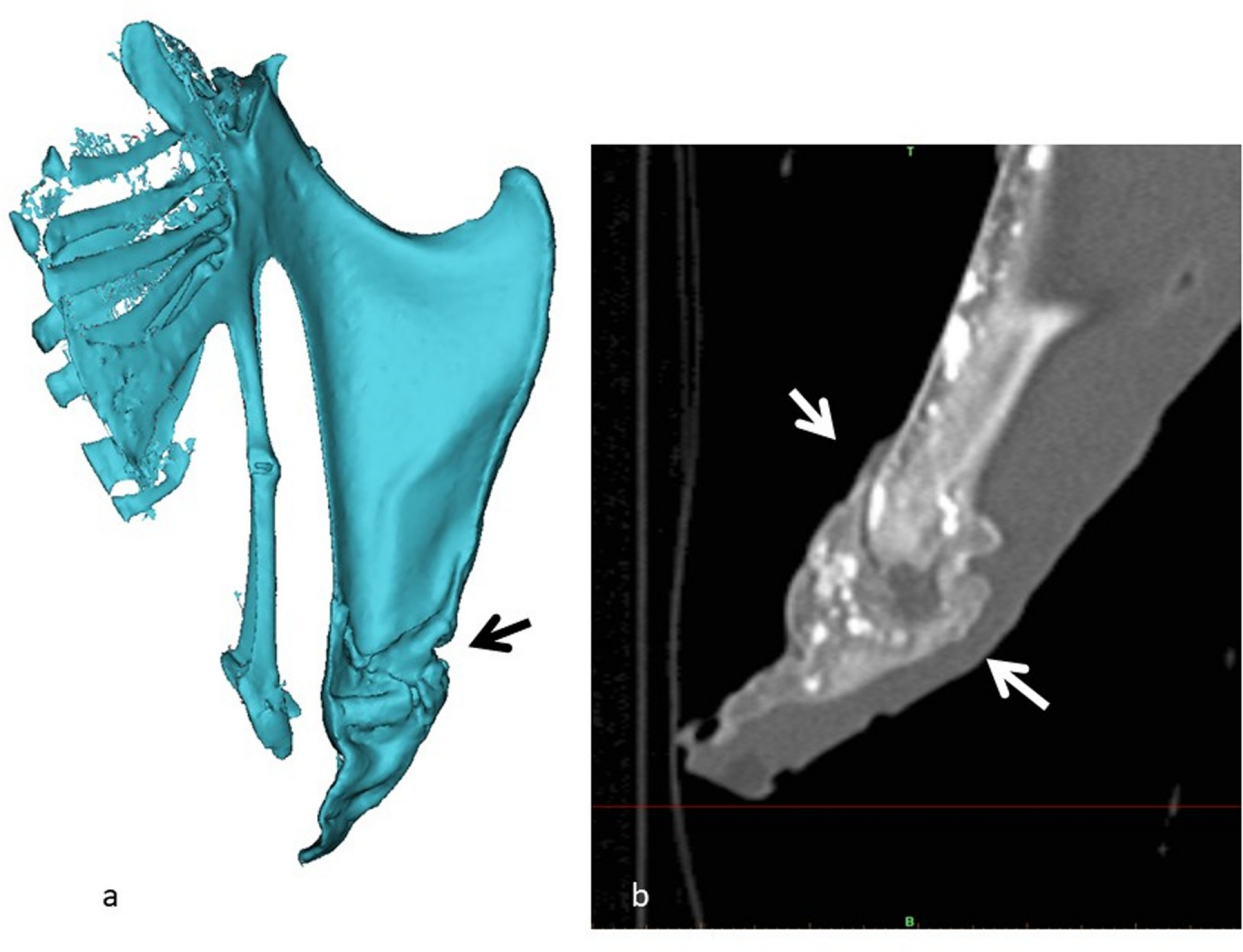
3D model (a) and CT image (b) of a 75+-week hen showing multiple fractures and the so-called pseudoarticulation (arrows).

**Fig 6:**
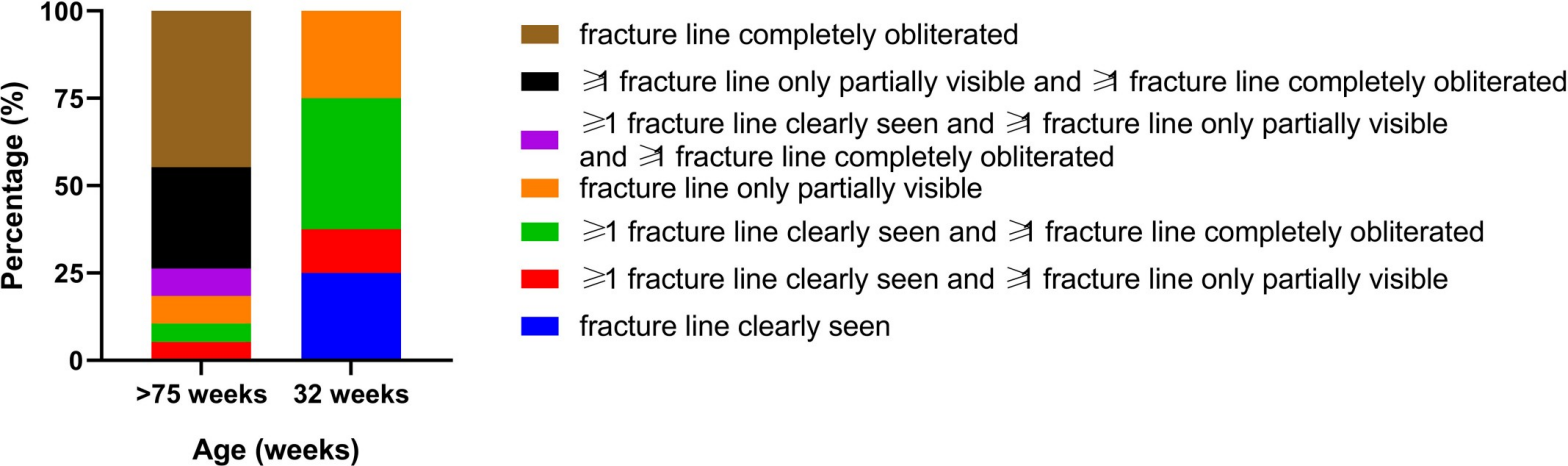
Age related distribution of remodeling status of the fracture lines identified by CT scan in old (n = 38) and young birds (n = 8). Data is presented as percentage within age group.

**Fig 7:**
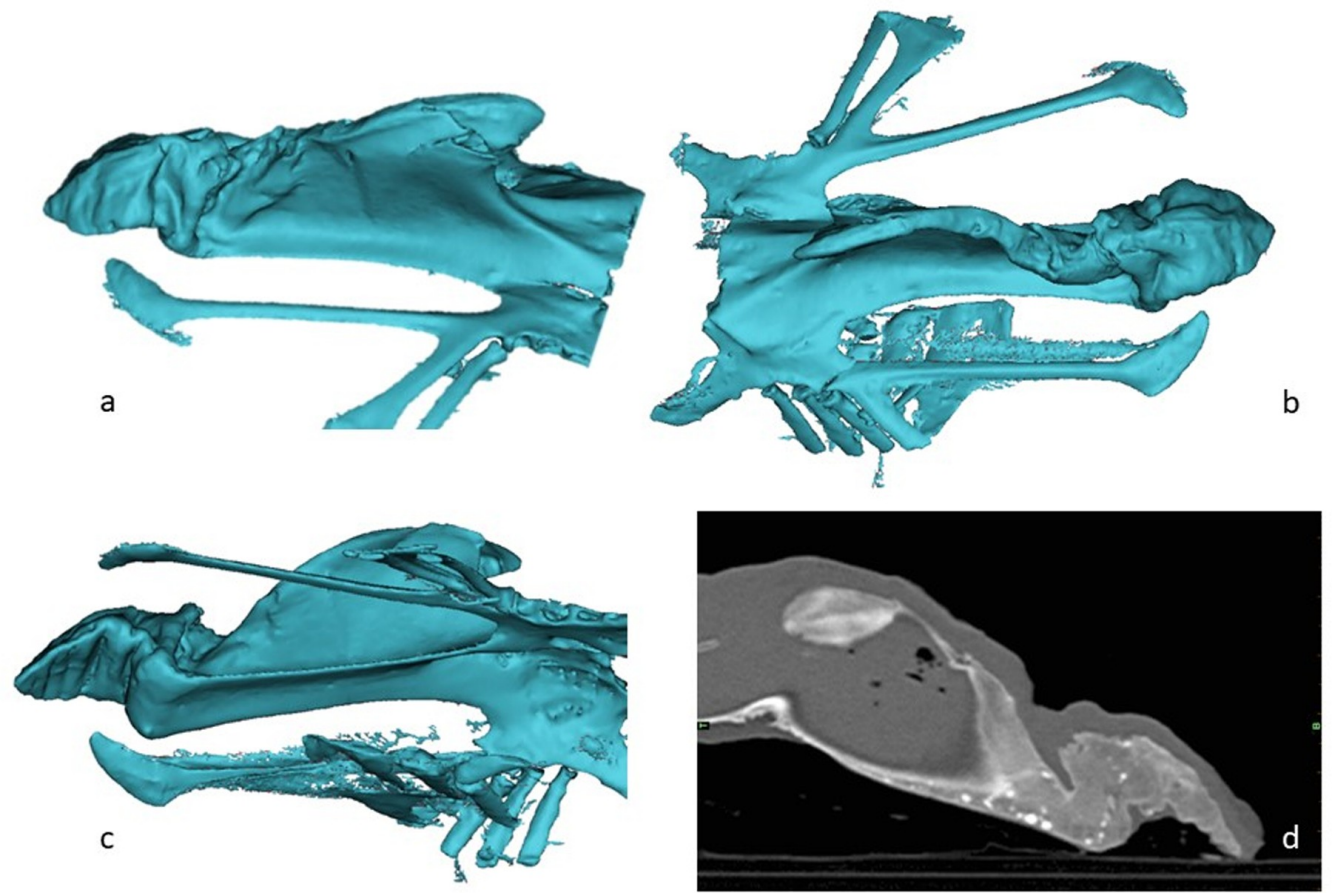
3D reconstruction (a,b,c,) and CT image (d) of a 75-week hen showing multiple fractures. Notice the radioopaque spots in the CT image.

Fractures was identified and characterized in 48 of the 62 keel bones (77%). In fourteen birds where no fracture was identified, five of these were the five birds that also presented no macroscopic fractures, the remaining nine birds were all >75 weeks old birds. The number of fractures in the birds with fractures differed between the two age groups: the proportion of birds that had one fracture was significantly higher in the 32 week old birds (Fig 3A and 3B), whereas the proportion of birds with >3 fractures (Fig 3C and 3D) was significantly higher in the older birds (z-test, P>0.05). Overall 44/48 (92%) of the fractures involved the caudal tip of the keel bone. In all the young birds with fractures, the fractures were located at the caudal part, 10/10 (100%) (Fig 4). In the old birds, 34/38 (89%) had fractures in the caudal part of the keel; half of these had also fractures in at least one the other two section of the keel. In Fig 5 the increase in localization complexity with increasing age is shown, however, the distribution is not significantly different. When it comes to remodeling state of the fracture lines there is a clear age related difference between the two age groups (Chi square; P<0.001) (Fig 6). This was demonstrated by the fact that clearly seen fracture lines (≥1 fracture line) were more frequently observed in the young birds, whereas completely obliterated fracture lines were only observed in the old birds (z-test, P>0.05). Finally, the CT scans revealed that in 25/49 of the old birds one or more radiopaque spots, i.e. points with highly increased radiographic density, (Fig 5D) and not in any of the young birds (Chi square, P = 0.001). These radiopaque spots were primarily observed in birds with substantial amounts of callus formation and/or deformation of the keel bone (23/25). Pseudo articulation in the caudal part of the keel bone was also observed in two birds with severe callus formation and deformation of the entire keel bone (Fig 7).

In the group of birds >75 weeks old (n = 49), CT scan was able to detect fractures in 12/14 birds housed in cages and 26/35 housed on deep litter, and no difference between the proportions in the ability to detect fractures by CT was observed (Chi square; P = 0.386). The proportion of caged birds presenting radiopaque spots (3/11) was significantly lower than in birds housed on deep litter (21/35) (Chi square; P = 0.015).

### Histology

#### Soft tissue damage

In general, no differences between the production systems concerning histopathological lesions were observed. Histological evaluation of the caudal half of the keel bone of 62 birds revealed minimal changes in the soft tissue (muscle and fascia) adjacent to the keel bone. Minimal hemorrhage was observed in four birds, hereof which three were old birds, with no age related difference (Chi square; *P* = 0.838). In two old birds minimal signs of inflammation was detected, with no age related difference (Chi square; *P* = 0.459). Minimal fibrosis was observed in seven old birds and in no young birds (Chi square; *P* = 0.148). In one young bird minimal muscular rupture was detected but not in old birds (Chi square; *P* = 0.050). Hemorrhage, inflammation, and muscular rupture were only observed in birds also identified with fracture, histologically. Fibrosis was observed in two birds without fractures and in five birds with fractures. No significant difference in the presence of soft tissue damage was observed between birds with fracture and birds with no fractures detected. No other damage to the soft tissue was observed.

#### Fracture morphology

In 38 out of 62 birds fracture lines were detected by histology in the caudal half of the keel bone. Of these, 31 were present in old birds and seven in young birds. The number of fractures in each birds was recorded (Table 3) and no age related difference was observed (Chi square; *P* = 0.783).

**Table 3:** Number of fractures observed in the caudal part of the keel bone by histological evaluation.

Age	No. of birds	No. of fractures						
		0	1	2	3	4	5	6
>75 weeks	49 (14^a^/35^b^)	18 (10/8)	20 (4/16)	5 (0/5)	3 (0/3)	2 (0/2)	0 (0/0)	1 (0/1)
32 weeks	13 ^b^	6	6	1	0	0	0	0

^a^ Caged birds.

^b^ Cage-free birds.

**Fig 8:**
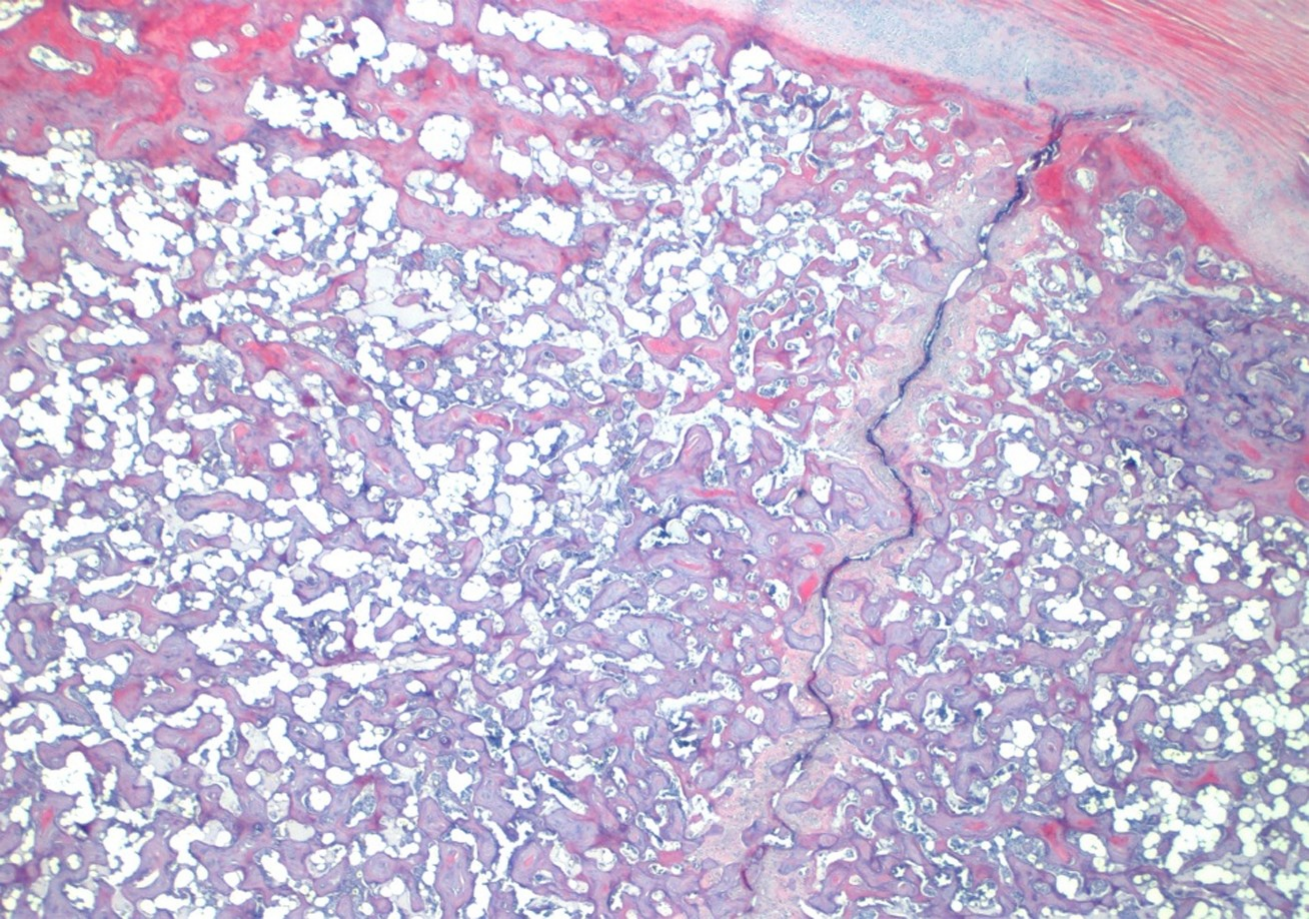
Typical un-branched fracture line with minimal repair processes (HE stain).

In 31 out of 38 birds, the fracture line appeared as a single un-branched line (Fig 8), in four birds the fracture line was branched and in two birds it was impossible to distinguish the fracture line appearance. No age related difference in this distribution was observed (Chi square; *P* = 0.753). In 29 birds the fracture line severed the periosteal lining at the ventral surface of the keel bone, in four birds the line opened up on the visceral (dorsal) surface, and in three birds the fracture was complete with severed the periost both ventrally and dorsally. No age related difference in this distribution was observed (Chi square; *P* = 0.764). In 32 birds, the fracture line was widest at the opening of the periost; in two birds, it was most narrow at the periosteal opening; in one bird, it was impossible to determine the widest point of the fracture line; and in two birds, the width of the fracture line was not recorded. Again no age related difference in the distribution was detected (Chi square; *P* = 0.718).

#### Fracture repair and inflammatory response

Hemorrhage in the fracture line was present in 6/38, 2/31 in old birds and 4/7 in young birds; although the degree of hemorrhage was minimal the proportion was significantly higher in the young birds (Table 4). Influx of inflammatory cells in the fracture line was absent in all birds. The proportion of birds without collagen in the fracture line was significantly higher in the young birds (6/7), whereas the proportion of birds with minimal presence of collagen in the fracture line was significantly higher in the old birds.

**Table 4:** Overview of inflammatory response and fracture repair in 38 birds with fracture lines in the caudal part of the keel bone observed by histological evaluation.

		Age ^1^		Total	*P*-value (Chi square)
		>75 weeks	32 weeks		
Fracture line—hemorrhage	Absent	29^a^	3^b^	32	0.001
	Minimal	2^a^	4^b^	6	
Fracture line—inflammation	Absent	31	7	38	N/A
Collagen in fracture line	Absent	12^a^	6^b^	18	0.058
	Minimal	13^a^	0^b^	13	
	Moderate	6	1	7	
Callus formation	Absent	8^a^	5^b^	13	0.063
	Minimal	17	1	18	
	Moderate	6	1	7	
Callus—immature	Absent	21	7	28	0.080
	Minimal	10	0	10	
Callus—mature (ossified)	Absent	17	5	22	0.715
	Minimal	8	1	9	
	Moderate	6	1	7	

^1^Different superscript letter denotes a subset of Age categories whose column proportions differ significantly from each other (z-test, *P*<0.05).

Callus formation was present in 25/38 birds. In the young birds the majority (5/7) showed no signs of callus formation, this proportion was significantly higher than in the old birds (8/31). Among the old birds 17 birds had minimal degree of callus formation, and six birds presented moderate amounts of callus. In the young birds, one bird showed minimal callus formation and one bird moderate amounts (*P =* 0.063, Table 4). However, when pooling all birds with presence of callus (minimal and moderate) within each age group the proportion of birds with at least minimal callus formation is significantly higher in the old birds (*P =* 0.0341, Fisher’s exact test). Immature callus was only present in the old birds (10/31), however no difference between the old and young birds was detected. Completely ossified (mature) callus was present in 16/38 birds. Among the old birds, eight birds had minimal degree of mature callus, and six birds presented moderate amounts of mature callus. In the young birds, one bird showed minimal mature callus and one bird moderate amounts (*P =* 0.715, Table 4). No difference between the old and young birds was detected, when pooling all birds with mature callus (minimal and moderate) within each age group (*P =* 0.6754, Fisher’s exact test)

The old birds originated from two different housing systems. The presence of fractures demonstrated by histology was significantly higher in birds housed on deep litter (27/35) than in caged birds (4/10) (Table 5). No difference in soft tissue damage and- fracture morphology was detected between housing systems (Table 5). No difference in level of fracture repair could be observed but there seems to be a trend towards more collagen and callus deposition in the birds housed on deep litter. However, this is only based on observations from four birds (Table 5).

**Table 5:** Overview of fracture repair in old birds from different housing types.

		Housing ^1^		Total	*P*-value (Chi square)
		Cages	Deep litter		
Fracture line (n = 49)	Absent	10^a^	8^b^	18	0.001
	Present	4^a^	27^b^	31	
Fracture line–inflammation (n = 31)	Absent	4	27	31	N/A
Collagen in fracture line (n = 31)	Absent	2	10	12	0.574
	Minimal	2	11	13	
	Moderate	0	6	6	
Callus formation (n = 31)	Absent	2	6	8	0.377
	Minimal	2	15	17	
	Moderate	0	6	6	
Callus–immature (n = 31)	Absent	3	18	21	0.739
	Minimal	1	9	10	
Callus—mature (ossified) (n = 31)	Absent	3	14	17	0.715
	Minimal	1	7	8	
	Moderate	0	6	6	

^1^Different superscript letter denotes a subset of Housing categories whose column proportions differ significantly from each other (z-test, *P*<0.05).

## Discussion

Quite a lot of research efforts in relation to keel bone fractures in the areas of diagnosing (by palpation, radiographs), genetics, feed components, productivity and welfare issues have been made. However, few, if any, investigations have addressed the perhaps most important question in order to understand the cause of this condition: what does the pathology of these fractures tell us? In order to suggest intervention measures for this condition it is pivotal to understand the nature of this condition.

It is striking how similar the fractures appear concerning localization and appearance. One aspect observed macroscopically was that the fractures in alternative systems generally are more productive often with substantial callus formation. In contrast, the fractures observed in caged birds were more discrete, often just a fine whitish line, making the fracture resemble a “greenstick” fracture or a stress fracture. From these observations it may be concluded that palpation as a means of diagnosing keel bone fractures in caged layers may be of little use. Consequently, it is strongly recommended that in order to estimate the true prevalence of keel bone fractures in caged birds post mortems or X-rays (when practically possible) should be carried out [24]. Furthermore, many fractures in birds from alternative systems also represent diagnostic problems in the field as their localization ventrally of the caudal part of the sternum makes them difficult to reach. The most common localization at the last 5 cm of the caudal part of the tip found in our material has been reported previously [26]. Also the furculum has been reported to be commonly affected [4]. Trauma as the cause of fracture makes sense in these prominent parts of the keel reported from some countries. This could theoretically be explained by differences in the housing systems. Some countries have used very high placed aerial perches (sometimes not fixed) which could promote impact injuries [5]. Such cranially located fractures resulting from external trauma often are relatively easy to palpate. However, the localization observed in our material points in another direction concerning the pathogenesis.

It can be speculated that the different morphology of the fractures observed in the cage system and the alternative systems is due to the differences in the birds’ ability to move around freely. Thus, these caudally located fractures may develop following a common pathogenesis e.g. due to the late ossification of the keel bone, but the mobility of the birds in alternative systems requires a lot of callus formation in order to try to immobilize and repair the fractures as previously described [33].

The hypothesis that the fractures develop due to high energy collisions was not supported in this study. If that was the case soft tissue damage in the form of hemorrhage, muscular rupture and subsequent fibrosis would be observed. In mammals including humans, soft tissue damage will develop if trauma causing fractures are applied and subsequent scarification/fibrosis leaving a fibrotic scar, which persists indefinitely, can be observed [34]. The healing processes in birds are generally believed to be similar to mammals and consequently we would expect to observe the same in birds [35]. In our material these lesions were rarely observed and no differences in the presence of soft tissue damage were observed between birds with fractures and birds without fractures. The appearance of the fracture lines did not support trauma as the cause of the fractures either. In the majority of the birds a single unbranched fracture line was observed. A complex branched fracture line or a comminuted fracture would be expected in case of a high impact trauma [36]. In case of high energy collisions it would be expected that the factures would be more open dorsally (viscerally), as the direction of the force would be front to back i.e. ventral to dorsal (visceral) causing the fracture line to “open” at the opposite (dorsal/visceral) side of the bone. In relation to fracture repair and inflammatory response it is striking how limited the healing process appear. Influx of inflammatory cells was absent in all birds which is characteristic for stress and greenstick fractures where a constant stress impedes healing. Greenstick fractures have been suggested previously [26] but this study is the first to show that the fractures indeed do resemble this type of fracture due to their histopathological appearance.

As previously demonstrated CT scanning is a useful research tool to study the morphology and the number of fractures and exact location [37]. CT scanning allows the investigation of the external appearance of the fractures thought the 3D models, as well accurate inspection of what happened inside the bone. This enables as to better evaluate the remodeled fractures.

A new type of high radiopaque points was observed by this method. This observation needs further investigations as it is not described in cadavers and bones before.

This investigation has shown that high energy collisions cannot be responsible for the majority of fractures, located at the caudal tip of the keel bone, observed in laying birds. However, only few other observations suggest alternatives to this commonly accepted theory. In this respect, one interesting characteristic of the fractures may be of value. The fracture lines are wider ventrally than dorsally indicating that the force initiating the fractures comes from the inside (visceral side of the keel bone). It may be speculated that earlier breeding goals including breeding for a smaller bird and a higher egg production may have resulted in some undesirable effects [38,4,39]. An increased egg production from a smaller metabolic mass has been speculated to result in depletion of the birds’ body reserves [40]. Subsequently, biomechanically there could be an effect of the egg laying process on the tip of the keel bone. This is supported by the appearance of the fracture lines indicating a pressure from the inside. However, forces from e.g. wing flapping may indeed also be a contributing factor. In addition to this, the late ossification of the caudal tip of the keel bone seems to represent a week spot in relation to the development of the fractures. Apparently, the ossification of the caudal tip of the keel bone (the area where fractures mainly are present) is not completed before the birds are 35–40 weeks old [41,42]. This is at the same time the fractures start to appear (at the age 26 to 30 weeks of age) and where egg production is high, which is a striking coincidence. Further to this it has been indicated that the development of fractures reduce after 45 weeks of age [14,26], again pointing at the late ossification as a possible inducer or risk factor for fractures. Although we were not able to demonstrate fractures directly in the ossification line, the appearance of the fractures from all production systems similar to greenstick fractures which are common in children and associated with the ossification zones [43], suggests this area of the keel for future research.

In conclusion, the fractures from cage-free and cage systems investigated in detail showed a similar appearance with absent soft tissue damage and minimal repair processes taking place. In addition, the fracture line was widest at the opening of the periost. This suggests a common pathogenesis where the lack of healing processes points at stress or poor ossification of the tip of the keel (greenstick fractures) as important factors in the development. Further, it appears as if the fractures develop from the inside by some yet unexplained mechanism.

## Supporting information

S1 Data (XLSX)
